# Knowledge about preventive dentistry versus self-reported competence in providing preventive oral healthcare – a study among Nepalese dentists

**DOI:** 10.1186/s12903-017-0366-5

**Published:** 2017-04-12

**Authors:** Madhu Wagle, Ganesh Acharya, Purusotam Basnet, Tordis A. Trovik

**Affiliations:** 1 Women’s Health and Perinatology Research Group, Department of Clinical Medicine, Faculty of Health Sciences, UiT - The Arctic University of Norway, N – 9037 Tromsø, Norway; 2 Department of Obstetrics and Gynaecology, University Hospital of North Norway, Tromsø, Norway; 3 Department of Clinical Science, Intervention and Technology, Karolinska Institute, Stockholm, Sweden; 4 Department of Community Medicine, Faculty of Health Sciences, UiT - The Arctic University of Norway, Tromsø, Norway

**Keywords:** Dentists, Preventive dentistry, Preventive knowledge, Oral health, Oral healthcare, Continuing Dental Education (CDE)

## Abstract

**Background:**

Dentists’ and dental healthcare providers’ professional knowledge and attitude towards the prevention of oral diseases may have an impact on the oral health of the general population. The aim of this study was to describe Nepalese dentists’ competency in giving preventive education and treatment to their patients, and to assess their level of knowledge about preventive dental health.

**Methods:**

This was a cross-sectional study of 195 dentists (71 males and 124 females). Knowledge of preventive oral healthcare and self-reported aspects of preventive oral healthcare were assessed using a close-ended multiple-choice questionnaire. Statistical evaluation was done using chi-squared test, independent sample t-test and factor analysis as appropriate.

**Results:**

More than 90% of dentists self-reported to be competent in providing preventive treatment and oral hygiene education to their patients. Female dentists reported being more competent in giving oral hygiene education than their male counterparts (*p* = 0.045). Dentists scored a mean of 24.06 ± 3.8 [range (15–33)] out of 56 on knowledge based on self-reported awareness of seven different aspects of preventive dentistry. More than 70% of the dentists had relatively good knowledge regarding the use of fluoride, whereas the preventive knowledge in other aspects of dental health such as frequency of sugar consumption, xylitol use, dental visits, sealant, gingival health, dental and general health was found to be limited.

**Conclusions:**

The majority of participating dentists reported a high level of general competency in providing preventive treatment and oral health education to their patients, whereas their knowledge was found to be limited in some aspects of preventive dentistry.

**Electronic supplementary material:**

The online version of this article (doi:10.1186/s12903-017-0366-5) contains supplementary material, which is available to authorized users.

## Background

The importance of preventing oral diseases for improving the general wellbeing of society is supported by the accumulated evidence showing an association between dental problems and a number of systemic diseases such as cardiovascular disease, coronary heart disease, diabetes, pneumonia and others [1–5]. Oral health is still not a national health priority, particularly in low and middle-income countries, and the prevalence of oral diseases has been increasing [6]. Some cultures ignore oral health, as teeth are considered expendable [7]. Additionally, in such countries, there are insufficient numbers of dentists and other dental healthcare personnel, and the availability of oral healthcare services is limited to densely populated cities [7]. Control of oral disease also depends heavily on individuals’ awareness of oral health and their accessibility to oral healthcare services [6]. Nonetheless, oral health might improve if services were focused on primary healthcare and prevention [8]. Some industrialized countries have successfully included preventive strategies in their oral health legislations, and a decline in one of the most prevalent oral disease worldwide, i.e. dental caries, has been reported [9–12]. Finally, prevention is essential to control and minimize oral and consequently systemic diseases [7, 13].

Oral health education and professional knowledge of preventive dentistry have enabled dentists to become role models for the general population [14–16]. Dental healthcare personnel can facilitate improvement in oral health due to their awareness and caring behaviour towards patients’ oral healthcare habits [17–19]. To achieve improved oral health in society, dental healthcare personnel are required to have abundant knowledge and a positive attitude, not only towards treatment, but also towards preventive oral healthcare [20–22]. A number of studies have assessed the knowledge of and attitude towards oral health among dental healthcare providers [14, 23–26]. Some studies have reported that dental healthcare personnel might not be fully updated on the effectiveness of preventive measures [27–31]. However, in the Nepalese context, systematic studies investigating this issue have not been reported.

The objective of our study was to describe Nepalese dentists’ (practising in Kathmandu) self-reported competency in giving preventive education and treatment, and to assess their level of knowledge about preventive dental health.

## Methods

This cross-sectional study was conducted in 2006 among dentists working in Kathmandu, the capital city of Nepal, and its close neighbourhoods. The total number of dentists registered with the Nepal Dental Association (NDA) at the time of survey was 319, of whom 195 (61%) were recruited to participate in the study. Dentists were contacted by telephone, informed about the study, and invited to participate. Appointments were made according to their convenience. The main tool used for data collection was a set of questions that had been used in a comparable target population previously [32]. The questionnaire survey was carried out by one of the authors (MW) who was trained in the use of this tool and its calibration at the University of Bergen, Norway [33]. Informed written consent was obtained from all the study participants. Ethical approval for conducting the study was obtained from the Nepal Health Research Council (NHRC) and the Nepal Dental Association (NDA).

### Questionnaire

The questionnaire consisted of a set of questions (49 in total, Additional file 1 - Questionnaire) on socio-demographic characteristics, oral hygiene habits, knowledge on different aspects of preventive procedures, satisfaction towards function and appearance of teeth, perception towards preventive dentistry and information about continuing dental education (CDE) activities. All knowledge and clinical skill information was self-reported. The questions regarding preventive knowledge (PK) were replicated from parts of a questionnaire used in a previous study [14, 32]. The close-ended questionnaire included detailed questions about knowledge to ensure balanced information on these subjects. Fourteen PK questions on different aspects of preventive dentistry assessed knowledge on use of fluoride, frequency of sugar consumption, xylitol use, sealant, dentist visits, gingival health and relation between oral health and general health. The questionnaire was pilot tested before use and a few modifications concerning clarification of the content were undertaken accordingly.

### Statistical analysis

Sample size calculation was performed using the Raosoft Inc. Sample size calculator [34]. As stated, 319 dentists were registered in NDA at the time of survey. We assumed professional preventive knowledge to be close to 50%, and using an absolute precision of 0.05 and 95% confidence level, the required number of participants was calculated to be 175. Due to the possibility of refusals to participate and dropouts, we recruited 195 dentists.

Data coding was done after checking the completeness of collected data and entering the information into a computer database. Analysis was performed using IBM SPSS statistics 22.0 statistical package. Frequency tables were made and comparisons between groups were performed using a χ^2^ (chi-squared) test for categorical variables and an independent sample t-test for parametric continuous variables. Information about Continuing Dental Education (CDE) activities was acquired by asking the participants about the number of courses attended, duration of the courses, specific field of dentistry covered by the courses and any perceived need for more CDE activity in a particular field of the dentistry. The CDE activity was registered as the sum of attendances to CDE activity within any field of dentistry (restorative dentistry, preventive dentistry, prosthodontics, oral surgery, orthodontics, and pedodontics) in the last two years.

Competency in giving oral health education (OHE) about preventive dental care and competency in giving preventive oral health treatment, were assessed using global questions with 4 answering options ranging from not at all competent (1) to very competent (4). The 14 preventive dental knowledge statements included different aspects of dentistry such as fluoride and its uses; the knowledge of detrimental effects of sugar consumption; action of xylitol and sealant; dentist visits and knowledge about gingivitis and general oral health. Answers were given in a 4-point Likert-scale ranging from strongly disagree (1) to strongly agree (4), and additionally a ‘do not know’ option (excluded in the analysis).

To clarify and simplify the content of preventive knowledge, dimensional analysis or principal components factor analysis (varimox rotation) was carried out. This was done to reduce the total number of different knowledge variables by grouping the variables with similar characteristics together. Factor analysis was performed to generate a correlation matrix of all the variables; the factors were extracted based on their correlation co-efficient and rotated to maximize the relationship between the variables and some of the factors [35]. Two statements, PK7 and PK8, were excluded from the analysis due to high “no-response” and “don’t know” response. The dimensional analysis reduced the 12 preventive knowledge items into 4 factors with eigenvalues 2.4 for *factor 1*, 2.0 for *factor 2*, 1.8 for *factor 3*, and 1.6 for *factor 4*. The factor scores were used as weights to produce four new variables, which were interpreted as referring to *general preventive oral health knowledge*, *knowledge about theory in preventive oral treatment*, *knowledge in use of fluorides*, and *acknowledging the importance of visiting a dentist regularly* [35] (Tables 4 and 5). A *p*-value of < 0.05 is considered statistically significant.

## Results

The study population consisted of 195 dentists, 71 (36%) males and 124 (64%) females. The mean work experience was 6.22 ± 7.1 years for male and 3.72 ± 3.5 years for female dentists (*p* < 0.05). About 54% of the dentists had less than 3 years of work experience. Five female dentists and one male dentist reported to be jobless. The number of participants educated outside Nepal was higher than those educated within the country, and the sum of attendances to any CDE was on average 2.5 ± 4.0. The details of baseline characteristics of dentists are presented in Table 1.

**Table 1:** Frequency distribution of participant’s characteristics in males and females (*n* = 195); mean (sd) and % (n)

Variables	Male (*n* = 71)	Female (*n* = 124)
Mean age in yrs. (sd), range 24–56	32.0 (7.3)	28.4 (3.9)^†^
Mean years in practice (sd), range 0.2–31.0	6.2 (7.1)	3.7 (3.5)^†^
Mean no. of CDE’s (sd)	3.4 (4.8)	2.0 (3.3)^†^
Place of education, % (n)		
Nepal	29.6 (21)	24.2 (30)
Abroad^a^	70.4 (50)	75.8 (94)
Sector of practice, % (n)		
Public	27.1 (19)	25.2 (30)
Public and private	27.1 (19)	21.0 (25)
Private	45.7 (32)	53.8 (64)

^a^Most frequently India and Bangladesh (males/females: 43/58% and 7/13%, respectively)

†*p* < 0.05

Table 2 shows self-reported competence in giving preventive education and treatment based on the global questions. Both female and male dentists reported high competence (96-99%). There was no significant difference regarding age groups, education site, sector of work, and satisfaction with appearance or function of own teeth. Female dentists reported higher competency (= *quite competent*) in giving preventive oral health education than male counterparts (*p* = 0.045).

**Table 2:** Self-reported competence in giving preventive education and treatment (n = 195). Possible answers: not at all-, not very-, quite-, very competent

	Quite competent	Very competent	SUM Q + V
Competency in giving preventive oral health education, % (n)			
Male Female	36.6 (26) 54.8 (68)	59.2 (42) 44.4 (55)	95.8 (68)^a^ 99.2 (123)
Competency in giving preventive oral health treatment, % (n)			
Male Female	46.5 (33) 50.0 (62)	50.7 (36) 46.0 (57)	97.2 (69)^b^ 96.0 (119)

^a^Significant difference between gender; *p* = 0.045. NS between age-groups, education site, sector of work, satisfaction with appearance or function of own teeth

^b^NS difference between gender, age-groups, education site, sector of work, satisfaction with appearance or function of own teeth

More male (69.0%) than female (56.5%) respondents answered all 14 questions about preventive dentistry. Whereas 31 and 43.5% male and female dentists, respectively, did not answer at all or answered *don’t know* to one or more questions. The mean answer score among respondents who answered all questions and those who did not was 1.72 (±0.28) and 1.79 (±0.26), respectively. There was no significant difference between males-females or among younger-older respondents. Table 3 illustrates the median scores for the 14 statements on knowledge regarding preventive dental health. The highest scores were registered for the statement PK2 ‘*using fluoridated toothpaste is more important than the brushing technique’* with the mean of 2.85 ± 0.6. This was followed by PK3 *fluoride is the most important factor for tooth susceptibility to decay* 2.25 ± 0.8. A significant difference in knowledge was seen between the male and female dentists regarding the effect of fluoridation of drinking water (PK4, *p* < 0.01) and in the association between dental health and general health (PK14, *p* < 0.05). Respondents answering *do not know* to the questions varied from 0.5% in PK6 and PK9, 8.2% in PK7 and 24.6% in PK8, while *no response* varied from 0% in PK1/PK2 to 8.2% in PK7.

**Table 3:** Level of knowledge of different aspects of preventive dental knowledge (PK’s) among the dentists in Kathmandu, by gender (Range 1–4; strongly disagree – disagree – agree - strongly agree; 5 = don’t know. High score reflect high level of knowledge. n=178, Median (range)

	Variables	Total^†^	Female	Male	*p*-value^††^
	Knowledge about fluoride				
PK1	Brushing teeth with fluoride toothpaste prevent tooth decay	2 (1–5)	2 (1–2)	2 (1–3)	0.84
PK2	Using fluoridated toothpaste is more important than the brushing technique to prevent caries	**3 (1–5)**	3 (1–4)	3 (1–4)	0.15
PK3	Fluoride is the most important factor for tooth susceptibility to decay	2 (1–5)	2 (1–4)	2 (1–4)	0.47
PK4	Fluoridation of the drinking water is an effective, safe, and efficient way to prevent dental caries*	2 (1–5)	2 (1–3)	2 (1–3)	**0.009**
PK5	It is beneficial to recommend fluoride tablets and/or topical fluorides for children in areas without a fluoridated water supply	2 (1–5)	2 (1–3)	2 (1–3)	0.34
	Knowledge about sugar				
PK6	The frequency of sugar-consumption has a greater role than the total amount of sugar consumed in causing caries	**1 (1–5)**	1 (1–3)	1 (1–2)	0.90
*PK7* ^*#*^	*Sugar-free chewing gum has a positive effect on dental health*	2 (1–5)	2 (1–3)	2 (1–3)	*0.15*
	*Knowledge about xylitol*				
*PK8* ^*#*^	*Xylitol is not only non-cariogenic, but also suppresses the growth of acidogenic bacteria in dental plaque*	2 (1–5)	2 (1–3)	2 (1–3)	*0.60*
	Knowledge about sealant				
PK9	Sealant is effective in prevention of pit and fissure caries in molars	2 (1–5)	2 (1–2)	2 (1–2)	0.33
	Knowledge about frequency of dental visit				
PK10	It is beneficial to visit a dentist for regular check-ups	**1 (1–2)**	1 (1–2)	1 (1–2)	0.42
	Knowledge on gingival health				
PK11	Regular brushing helps in prevention of gum problems	**2 (1–3)**	2 (1–3)	1 (1–3)	0.67
PK12	Gingivitis is caused by dental plaque	2 (1–5)	2 (1–3)	2 (1–2)	0.46
PK13	Gingivitis can be cured by effective oral hygiene	2 (1–5)	2 (1–3)	2 (1–3)	0.69
	Knowledge on dental and general health				
PK14	Having dental problems can lead to general health problems*	2 (1–5)	2 (1–3)	2 (1–3)	**0.046**
	MEDIAN all answers (PK1-PK14)		1.929 (1–4)	1.857 (1–4)	

^†^Missing answers excluded (*n* = 17)

^††^ *p*-value-t-test for differences between genders

*p < 0.05

^#^ excluded during factor analysis due to large number of missing response

High score reflect high level of knowledge). *n* = 178, Median (range)

With principle components factor analysis of the 12 preventive knowledge items, the reduced four factors solution explained 58.3% of the total variance and 18.2%, 16.4%, 12.0% and 11.8% explained variances for Factor 1, Factor 2, Factor 3 and Factor 4, respectively (Table 4). Preventive knowledge statements 1, 6, 11, 12, 13 and 14 loaded primarily on the first factor ‘general knowledge’. Statement 4, 5 and 9 loaded primarily on the second factor ‘theoretical knowledge’. Statement 2 and 3 loaded primarily on the third factor ‘fluoride knowledge’, while statement 10 loaded only on the fourth factor ‘dentist visit’ (Table 4).

**Table 4:** Factor analyses (Varimax rotation) of 12 dental health preventive knowledge statements (score 1–4; low-high knowledge) with mean scores (sd) and coefficients that relate the variables to the four rotated factors; general preventive oral health knowledge, theory in preventive oral treatment, knowledge in use of Fluorides, and acknowledging the importance of visiting a dentist regularly

			Factor coefficients				
PK item^a^	n	Mean (sd)	General knowledge Factor 1	Theoretical knowledge Factor 2	Fluoride knowledge Factor 3	Visit dentist Factor 4	Communality
12	168	1.55 (0.51)	**0.781**	0.219	−0.109	0.059	0.673
13	168	1.57 (0.56)	**0.750**	0.054	0.125	−0.078	0.586
1	168	1.62 (0.50)	**0.561**	0.213	0.302	0.150	0.474
11	168	1.54 (0.53)	**0.519**	0.024	−0.073	0.469	0.496
6	168	1.42 (0.51)	**0.423**	0.446	−0.259	0.372	0.583
14	168	1.76 (0.53)	**0.409**	0.277	0.288	0.210	0.371
5	168	1.74 (0.54)	0.110	**0.804**	0.080	−0.069	0.670
4	168	1.77 (0.49)	0.159	**0.733**	0.047	0.064	0.568
9	168	1.54 (0.50)	0.131	**0.617**	0.099	0.348	0.528
2	168	2.85 (0.61)	−0.028	0.009	**0.790**	−0.149	0.647
3	168	2.25 (0.76)	0.161	0.116	**0.713**	0.239	0.604
10	168	1.23 (0.42)	0.008	0.115	0.097	**0.880**	0.798

^a^PK Statements in full text in Table 2

PK 7 and PK 8 excluded in Factor analyses

Bold numbers correspondes to the significant numbers

On an individual level, the majority of respondents (75.4%) reported Factor 3 to be the statements within which they had the best knowledge (mean score = 3.0, Table 5). Eighty-five percent of the respondents loaded high on one specific Factor only. Very few reported Factor 1 (general knowledge) and 2 (knowledge in prevention theory) to be their most knowledgeable topics (1 and 2%, respectively). A total of 5.6% of the respondents reported Factor 4 to represent their best knowledge (mean score = 2.0), although this question also had the highest number of non-responders (*n* = 59). There were no significant differences in response based on sex, dichotomized age groups (young and older) or the place of education of the dentists. However, dentists working in the private sector reported significantly more positive to Factor 3 and 4.

**Table 5:** Analysis of the 4 new Factors after Factor analyses (Varimax rotation) of 12 dental health preventive knowledge statements, mean (sd) score in each Factor, proportion of respondents who loaded highest on the individual Factors

		Factors		
PK statements^a^	General knowledge Factor 1	Theory Factor 2	Fluoride knowledge Factor 3	Visit dentist Factor 4
Missing	18	10	7	**59**
Mean per statement (sd)	1.58 (0.34)	1.69 (0.38)	**2.53 (0.55)**	1.95 (0.44)
Range	1.33	1.33	3.00	2.00
Respondents with only 1 factor loading^b^ n (%)	2 (1.0)	4 (2.1)	**147 (75.4)**	11 (5.6)
Bivariate significant difference				
Sex Age Educated in Nepal/Abroad Sector (priv./publ.)	NS NS NS NS	NS NS NS NS	NS NS NS ***p*** **= 0.042**	NS NS NS ***p*** **= 0.009**

^a^PK Statements in full text in Table 2

^b^84.5% of the respondents loaded highest on one specific Factor. Most frequently, when several Factors loaded equally: Factor 3 + 4: 13 (6.7%); Factor 2 + 3 + 4: 9 (4.6%)

Bold numbers correspondes to the significant numbers

## Discussion

As described by previous reports, most oral diseases remain untreated in low-income nations due to limited financial and dental manpower resources [36]. Nepal is among one of the poorest and least developed nations in the world. For most people, the cost of regular dental treatment will be too expensive. It is therefore important that more emphasis should be placed upon prevention rather than on treatment of oral diseases. Moreover, for the provision of preventive dental treatment, the knowledge of the significance of preventive measures is of utmost importance. With relevance to this, our study has indicated that despite having relatively good knowledge in some aspects of preventive oral health, and positive self-reported competency in giving preventive treatment and providing OHE to their patients, Nepalese dentists’ knowledge in other areas of preventive dentistry is limited.

The common possible and viable approaches to prevent dental diseases involve removal of plaque, reduction in sugar consumption, and increasing the tooth’s resistance to the effects of acid. Since dental plaque is the main cause for most prevalent oral diseases such as ‘dental caries’ and ‘periodontal diseases’, knowledge about plaque and gingival health is of fundamental importance (PK11-13). Remarkably, these questions were answered with “disagree” (=2) and even strongly disagree (=1). Since these are statements on the generally accepted association between dental health and its preventive tools, this raises a reason for concern. E.g., that the mean answering score to the statement “*the frequency of sugar-consumption has a greater role than the total amount of sugar consumed in causing caries*” (PK6) was 1 (= strongly disagree) is not easy to explain. It is further discouraging that 10% demonstrated lack of knowledge of sugarless chewing gum (PK7), although the use of gum is widespread in Nepal [37, 38].

Fluoride has been described as the main factor responsible for the decline in the prevalence of dental caries worldwide [39]. Although reports state that the fluoride concentration in groundwater in Kathmandu is within the level recommended by WHO, the water is not directly drinkable due to the presence of excessive amounts of iron and bacteria. Consequently, there is no reason to believe that most people are supported with sufficient levels of fluoride from drinking water [40]. That almost one third of study participants disagreed with the statement that use of fluoride toothpaste is more important than the brushing technique for caries prevention (PK2), is in line with findings among Iranian and Mongolian dentists [15, 24, 32]. The general lack of knowledge about toothpaste fluoride (PK1) could be due to traditions, cultural differences, or study curriculum in educational institutions [32].

Similarly, one third of the dentists did not know whether xylitol have an anti-cariogenic function or not, or refused to answer this question (PK8). This is in line with a recent review of 10 randomised controlled trails, including almost 6000 participants, reporting low to very low quality evidence of effectiveness of xylitol in the prevention of dental caries, whether used in toothpaste containing fluoride or in any other supplementary form [41]. It nonetheless gives reason for concern. Xylitol is a naturally occurring sweetener, and albeit there is a mixed controversy regarding its effectiveness in dental caries reduction, previous studies have reported it to be effective in reducing the incidence of dental caries, and equally effective whether used as a dietary sugar supplement or in chewing-gum [42].

The general preventive knowledge factor for the Nepalese dentists seemed to be low when compared to other factors such as fluoride knowledge, importance of dental visits and theoretical aspects of preventive knowledge. It is also surprising to recognise that only approximately 6% of the dentists acknowledged the importance of regular dental visits, while a large number of the participants refused to give answers.

Presence of bias on different levels is a challenge for most observational studies. “Social desirability bias” could explain why a relatively high number of dentists agreed to participate in the study when invited. Dentists may have over-reported the global “good behavior” questions to give answers in a socially acceptable direction and present a favorable image of themselves [43]. “Information bias” might be present, as questions could have been misinterpreted, under or over-reported, although the pilot indicated that *face validity* seemed to be satisfactory [44, 45]. The questionnaire used has been validated previously in a similar population, but in a different cultural setting [32]. This could also be one of the reasons for the differences in response to similar questions. The question of what the most appropriate and robust method for evaluating preventive knowledge is among dentists, remains unresolved.

One other limitation of our study is that only dentists working in Kathmandu valley were included and therefore the generalizability of the study’s results may be questionable. However, our study population represented 61% of all dentists registered with the NDA (*n* = 319) at the time of this survey (2006). That the majority of participating dentists were of a younger age and with rather short clinical experience could be suggestive of their interest and awareness regarding the importance of oral healthcare. The preventive care knowledge of dentists may decline over a time period after graduation, whereas recent graduates are known to have more knowledge on prevention [27] and a positive attitude towards preventive care related practices [46].

## Conclusions

The majority of the Nepalese dentists who participated in this survey reported high general competency in giving preventive treatment and oral health education to their patients, but a detailed investigation into preventive knowledge revealed the possibility of general knowledge shortcomings, irrespective of a possible presence of bias. This indicates that updating dentists’ knowledge of preventive measures may be beneficial to promoting oral health in Nepal.

## Additional file

Additional file 1: Dentists' Questionnaire. (PDF 46 kb)

## Abbreviations

CDE: Continuing dental education
DMFT: Decayed, missing filled teeth
NDA: Nepal Dental Association
NHRC: Nepal Health Research Council
OHE: Oral health education
WHO: World Health Organization
