# Supplementary material for: Knowledge about preventive dentistry versus self-reported competence in providing preventive oral healthcare – a study among Nepalese dentists
Source: BMC Oral Health. 2017 Apr 12;17:76. doi: 10.1186/s12903-017-0366-5 (PMC5389144; doi:10.1186/s12903-017-0366-5)

ENCLOSURE 2
ID NO. ………..

DENTISTS' QUESTIONNAIRE

Personal data

(1) Year of birth _______

(2) Gender

1 ☐ Female
2 ☐ Male

(3) Year of graduating from dental school _______

(4) How long have you been working as a dentist________________________________

(5) Where did you complete your basic degree as a dentist?

1 ☐ In Nepal
2 ☐ Abroad (Specify) ____________________

(6) Current job(s)
(*You can tick more than one answer*)

1 ☐ Self employed dentist in private clinic
2 ☐ Employed dentist in private clinic
3 ☐ Dentist in public clinic
4 ☐ Dentist or specialist in hospital or dental centre
5 ☐ No clinical work presently

(7) Field of practice_______________________

(8) Patient group you presently work with
(*Please tick one only*)

1 ☐ General dentist
2 ☐ Children (0-14 yrs.)
3 ☐ Restorative/conservative dentistry
4 ☐ Adults
5 ☐ Prosthodontic dentistry
6 ☐ Mixed
7 ☐ Orthodontic dentistry
8 ☐ Oral surgeon
9 ☐ Paedodontic dentist
10 ☐ Periodontic dentistry

Oral hygiene and dietary behaviour and utilization of dental services

(9) How often do you usually brush your teeth?
(*Please tick one only*)

1 ☐ Once daily
2 ☐ Twice daily
3 ☐ Thrice daily
4 ☐ Once a week

10) How often do you clean the space between the teeth?
(*Please tick one only*)

1 ☐ Not every day
2 ☐ Once a day
3 ☐ Twice a day
4 ☐ After every meal
5 ☐ Never

(11) At what time do you primarily clean your teeth?
(*You can tick more than one answer*)

1 ☐ In the morning (without breakfast)
2 ☐ After breakfast
3 ☐ Usually before meals
4 ☐ Usually after meals
5 ☐ When going out
6 ☐ Before going to bed
7 ☐ Other times, (please specify) when

…………………………………………………

(12) Do you use toothpaste containing fluoride while brushing?

1 ☐ Always or almost always
2 ☐ Quite often
3 ☐ Seldom
4 ☐ Not at all
5 ☐ I use something else (please specify)

…………………………………………………..

(13) How often do you take meals during a day?

1 ☐ One time
2 ☐ Two times
3 ☐ Three times
4 ☐ Four times
5 ☐ Five times or more

(14) How often do you eat sugar-containing snacks between your main meals?

1 ☐ About 3 times a day or more
2 ☐ About twice a day
3 ☐ About once a day
4 ☐ Occasionally; not every day
5 ☐ Rarely or never eat between meals

(15) How often do you eat sugar-containing coffee between your main meals?

1 ☐ About 3 times a day or more
2 ☐ About twice a day
3 ☐ About once a day
4 ☐ Occasionally; not every day
5 ☐ Rarely or never drink between meals

(16) How often do you eat sugar-containing tea between your main meals?

1 ☐ About 3 times a day or more
2 ☐ About twice a day
3 ☐ About once a day
4 ☐ Occasionally; not every day
5 ☐ Rarely or never drink between meals

(17) About how long ago was you're most recently dental treatment?

1 ☐ No more than 6 month ago
2 ☐ More than 6 month up to 1 year ago
3 ☐ More than 1 up to 2 years ago
4 ☐ More than 2 years up to 5 years ago
5 ☐ More than 10 years ago
7 ☐ Never

(18) What was the reason for this visit?

1 ☐ I had trouble with my teeth/gums
2 ☐ For regular check-up
3 ☐ Other, please specify the reason

(19) How would you describe the condition of your mouth and teeth?

1 ☐ Good
2 ☐ Quite good
3 ☐ Average
4 ☐ Quite bad
5 ☐ Bad
6 ☐ I do not know

(20) In general, are you satisfied or dissatisfied with the appearance of your teeth?

1 ☐ Satisfied
2 ☐ Neither/nor
3 ☐ Dissatisfied

(21) In general are you satisfied or dissatisfied with the function of your teeth?

1 ☐ Satisfied
2 ☐ Neither/nor
3 ☐ Dissatisfied

Continuing Dental Education

(*This section has one question and two statements to be answered.)*

(22) If you had any learning opportunities during the last 2 years (2004-2005), in which field of dentistry, how many times, and how long did they take? Please tick whether you had any need for a continuing education course on these subjects regardless of whether you have taken a course on it during the last 2 years.

Fields	How many times?
1 Restorative	
2 Prosthodontics	
3 Oral surgery	
4 Prevention	
5 Pedodontics	
6 Orthodontics	

	How many days in total (estimated as working days)
1 Restorative	
2 Prosthodontics	
3 Oral surgery	
4 Prevention	
5 Pedodontics	
6 Orthodontics	

	Have you had need for any more courses? yes	no
1 Restorative	1 ☐	2 ☐
2 Prosthodontics	1 ☐	2 ☐
3 Oral surgery	1 ☐	2 ☐
4 Prevention	1 ☐	2 ☐
5 Pedodontics	1 ☐	2 ☐
6 Orthodontics	1 ☐	2 ☐

How do you agree to the following statements?

(23) Continuing education courses would be of great use for dentists

1 ☐ Strongly agree
2 ☐ Agree
3 ☐ Disagree
4 ☐ Strongly disagree
5 ☐ Don't know

(24) Continuing education should be compulsory for dentists

1 ☐ Strongly agree
2 ☐ Agree
3 ☐ Disagree
4 ☐ Strongly disagree
5 ☐ Don't know

Competency and orientation in preventive care

(Please tick one answer that mostly reflects your view.)

(25) In your opinion, having good teeth depends on:

1 ☐ By chance
2 ☐ Hereditary factors
3 ☐ Taking good dental care
4 ☐ Both (2+3)
5 ☐ I do not know

(27) How competent do you feel in giving preventive treatment to patients?

1 ☐ Very
2 ☐ Quite
3 ☐ Not very
4 ☐ Not at all

(28) How competent do you feel in giving oral heath education to patients?

1 ☐ Very
2 ☐ Quite
3 ☐ Not very
4 ☐ Not at all

How do you agree to the following statement?

(29) Training on and practice of preventive dentistry both in dental undergraduate education and in clinical dental practice should be increased

1 ☐ Strongly agree
2 ☐ Agree
3 ☐ Disagree
4 ☐ Strongly disagree
5 ☐ Don't know

Preventive knowledge

(30) Brushing teeth with fluoride toothpaste prevent tooth decay

1 ☐ Strongly agree
2 ☐ Agree
3 ☐ Disagree
4 ☐ Strongly disagree
5 ☐ Don't know

(31) Using fluoride toothpaste is more important than the brushing technique to prevent caries

1 ☐ Strongly agree
2 ☐ Agree
3 ☐ Disagree
4 ☐ Strongly disagree
5 ☐ Don't know

(32) Fluoride is the most important factor for tooth susceptibility to decay

1 ☐ Strongly agree
2 ☐ Agree
3 ☐ Disagree
4 ☐ Strongly disagree
5 ☐ Don't know

(33) Fluoridation of the drinking water is an effective, safe, and efficient way to prevent dental caries

1 ☐ Strongly agree
2 ☐ Agree
3 ☐ Disagree
4 ☐ Strongly disagree
5 ☐ Don't know

(34) It is beneficial to recommend fluoride tablets and/or topical fluorides for children in areas without a fluoridated water supply

1 ☐ Strongly agree
2 ☐ Agree
3 ☐ Disagree
4 ☐ Strongly disagree
5 ☐ Don't know

(35) The frequency of sugar-consumption has a greater role than the total amount of sugar consumed in causing caries

1 ☐ Strongly agree
2 ☐ Agree
3 ☐ Disagree
4 ☐ Strongly disagree
5 ☐ Don't know

(36) Sugar-free chewing gum has a positive effect on dental health

1 ☐ Strongly agree
2 ☐ Agree
3 ☐ Disagree
4 ☐ Strongly disagree
5 ☐ Don't know

(37) Xylitol is not only non-cariogenic, but also suppresses the growth of acidogenic bacteria in dental plaque

1 ☐ Strongly agree
2 ☐ Agree
3 ☐ Disagree
4 ☐ Strongly disagree
5 ☐ Don't know

(38) Sealant is effective in prevention of pit and fissure caries in molars

1 ☐ Strongly agree
2 ☐ Agree
3 ☐ Disagree
4 ☐ Strongly disagree
5 ☐ Don't know

(39) It is beneficial to visit a dentist for regular check-ups

1 ☐ Strongly agree
2 ☐ Agree
3 ☐ Disagree
4 ☐ Strongly disagree
5 ☐ Don't know

(40) Regular brushing helps in prevention of gum problems

1 ☐ Strongly agree
2 ☐ Agree
3 ☐ Disagree
4 ☐ Strongly disagree
5 ☐ Don't know

(41) Gingivitis is caused by dental plaque

1 ☐ Strongly agree
2 ☐ Agree
3 ☐ Disagree
4 ☐ Strongly disagree
5 ☐ Don't know

(42) Gingivitis can be cured by effective oral hygiene

1 ☐ Strongly agree
2 ☐ Agree
3 ☐ Disagree
4 ☐ Strongly disagree
5 ☐ Don't know

(43) Having dental problems can lead to general health problems

1 ☐ Strongly agree
2 ☐ Agree
3 ☐ Disagree
4 ☐ Strongly disagree
5 ☐ Don't know

General questions

(44) Is maintaining teeth important to you?

1 ☐ Very important
2 ☐ Quite important
3 ☐ Average
4 ☐ It is not important
5 ☐ I do not care

(45) What do you use to clean the space between the teeth?

1 ☐ Toothpick
2 ☐ Dental floss
3 ☐ Interdent brush
4 ☐ Nothing

(46) Where did you have dental treatment? (If have any) *Please tick one only*

1 ☐ Hospital
2 ☐ own clinic
3 ☐ Private dental clinic

(47) If you have not had dental treatment for two years, what is the reason?
(*You may choose more than one answer*)

1 ☐ I did not have any problems
2 ☐ Dental care is unpleasant
3 ☐ Dental care is too expensive
4 ☐ I did not have time
5 ☐ Other reasons (please specify)

………………………………………………………

(48) Do you currently experience, or have you earlier experienced, gingival bleeding?

1 ☐ Currently experiencing gingival bleeding
2 ☐ Earlier experienced gingival bleeding, but not now
3 ☐ Never had gingival bleeding

(49) Do you currently experience tooth mobility because of periodontal problems?

1 ☐ No
2 ☐ Yes

THANK YOU

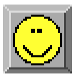

Supplement: Additional file 1: — Dentists' Questionnaire. (PDF 46 kb) [file 12903_2017_366_MOESM1_ESM.pdf]
